# Thymol Encapsulated into HP-β-Cyclodextrin as an Alternative to Synthetic Fungicides to Induce Lemon Resistance against Sour Rot Decay

**DOI:** 10.3390/molecules25184348

**Published:** 2020-09-22

**Authors:** Vicente Serna-Escolano, María Serrano, Daniel Valero, María Isabel Rodríguez-López, José Antonio Gabaldón, Salvador Castillo, Juan Miguel Valverde, Pedro Javier Zapata, Fabián Guillén, Domingo Martínez-Romero

**Affiliations:** 1 Department of Food Technology, University Miguel Hernández (UMH), Ctra. Beniel km. 3.2, Orihuela, 03312 Alicante, Spain; vicente.serna02@goumh.umh.es (V.S.-E.); daniel.valero@umh.es (D.V.); scastillo@umh.es (S.C.); jm.valverde@umh.es (J.M.V.); pedrojzapata@umh.es (P.J.Z.); fabian.guillen@umh.es (F.G.); 2 Department of Applied Biology, University Miguel Hernández, Ctra. Beniel km. 3.2, Orihuela, 03312 Alicante, Spain; m.serrano@umh.es; 3 Departamento de Ciencia y Tecnología de Alimentos, Universidad Católica San Antonio de Murcia (UCAM), Avenida de los Jerónimos s/n, 30107 Guadalupe, Murcia, Spain; mirodriguez@ucam.edu (M.I.R.-L.); jagabaldon@ucam.edu (J.A.G.)

**Keywords:** *Citrus limon*, postharvest, cyclodextrin, *Geotrichum citri-aurantii*, incidence decay, severity decay

## Abstract

Consumers demand the use of eco-friendly fungicides to treat fruit and vegetables and governmental authorities have unauthorized the application of chemical antifungals for the efficient control of sour rot. In the present research, the microwave irradiation (MW) method was used to encapsulate thymol into 2-hydroxylpropyl-beta-cyclodextrin (HP-β-CD) and the effect of these HP-β-CD on controlling sour rot in citrus fruit, caused by *Geotrichum citri-aurantii*, was evaluated. Amounts of 25 and 50 mM of HP-β-CD-thymol were used, and compared with propiconazole, to control the decay of inoculated lemon fruit. The treatments were performed in curative and preventive experiments. The incidence and severity of *Geotrichum citri-aurantii* in 25 and 50 mM HP-β-CD-thymol-treated fruit were reduced in both experiments. The preventive 50 mM HP-β-CD-thymol treatment showed the best effect, reducing the sour rot, respiration rate and fruit weight loss during storage at 20 °C. HP-β-CD-thymol increased polyphenol concentration and the activity of antioxidant enzymes, such as catalase (CAT), ascorbate peroxidase (APX) and peroxidase (POD) in lemon peel, and the highest effects were found with the 50-mM dose. In conclusion, the results show that the use of thymol encapsulated by MW into HP-β-CD could be an effective and sustainable tool, a substitute to the synthetic fungicides, for *G. citri-auriantii* control in citrus fruit.

## 1. Introduction

Sour rot disease, occasioned by the fungus *Geotrichum citri-aurantii* (*G. citri-aurantii*), is one of the main rot types of citrus fruit [1]. This decay is the second cause of post-harvest citrus decay after *Penicillium* spp. [2]. *G. citri-aurantii* poses serious problems for its control and eradication [3] because it is present in the soil and it is dispersed by air, by splashing rainwater and irrigation towards the surface of the fruit. The pathogen enters the fruit throughout wounds on the skin caused by mechanical damage, wind or insects during on-tree fruit development, harvesting or postharvest handling, and once the fungus entry to the albedo the disease begins [4,5]. *G. citri-aurantii* involves the secretion of extracellular endopolygalacturonases (PG) which quickly macerate the tissues of the fruit and the contact between fruit cells and the secreted juices of the infected area can reach the healthy areas propagating the rot during storage [2]. The ideal conditions for infection to occur are turgid fruit skin with free water, ambient temperatures between 12 and 36 °C, pH ranging from 2.0 to 8.5, high relative humidity (92% to 98%) and availability of sugars, acids and starch. In addition, the aggressiveness of the fungus increases over degreening and fruit maturation [3].

The traditional treatment to avoid fungal diseases has been the use of synthetic fungicides. However, nowadays there are only a few available fungicides against *G. citri-aurantii* since most of the fungicides used to control other fungal diseases are ineffective against this fungus, have created resistance or have been restricted by administrative authorities, due to different environmental and health problems related to the accumulation of residues in food. For instance, guazatine [3] was very effective for managing sour rot, but its use has been banned in different countries (European Union or United States) due to its risks on human health. Propiconazole is also an effective fungicide and authorized by the European Union against *G. citri-aurantii*. However, the use of this fungicide was allowed just until March 2020 in European Union because it is a systemic triazole fungicide with a high environmental risk [6].

Some strategies have been used to control of *G. citri-aurantii*, such as chlorine [7], gaseous ozone or ozonated water [8], exposures to elevated CO_2_ concentration [9]. In addition, some inorganic salts which are incorporated in the generally recognized as safe compounds list (GRAS), has also been proved to be effective, the most important being sodium bicarbonate and potassium sorbate [1], the antagonists microorganism *Bacillus subtilis* [10], *Muscodor albus* [5], *Rhodosporidium paludigenum*, *Cryptococcus laurentii* [4], *Pichia pastoris* [11] and *Cytosporone* B [12], plant extract from *Cistus villosus* and *Halimium antiatlanticum* [13] and essential oils from plant species [14].

*Thymus* spp. essentials oils [15] or thymol as pure compounds [16] have been reported to act as naturals fungicides when applied directly on vegetables. However, these strategies to control *G. citri-aurantii* are not totally effective or can cause fruit alteration, such as phytotoxicity, off flavor and odd tastes and aromas [17]. On the other hand, it is necessary to find strategies for essential oil application aimed to surpass the disadvantages derived from the physicochemical properties of thymol or thyme oil, such as their low palatability due to its distasteful and potent smell, low water solubility and unsteadiness due to oxygen, light and temperature, all of them limiting their use in postharvest fruit industry [18].

Encapsulation of essential oils in different types of cyclodextrins (CD) is a technique that releases the essential oils for long periods of time, avoids their degradation, improves their solubility [19] and reduces aroma compound intensity [20]. Nevertheless, it is necessary to choose the most suitable CD and encapsulation methods depending on solubility of the encapsulated compound and the agro-food applications. 2-hydroxylpropyl-beta-cyclodextrins (HP-β-CDs) showed higher aqueous solubility and stability than α- and β-CDs [21]. Moreover, Rodríguez-López et al. [18] showed that the best encapsulation efficacy of thymol in CDs was reached in HP-β-CDs compared with α- and β-CDs and creating very stable complexes with microwave (MW) encapsulation method, which was demonstrated by the high affinity of HP-β-CDs for thymol molecules. In this way, it is possible to reduce the negative effects of pure essential oils when they are applied directly on fruit surface and, in addition, their effectiveness against microorganisms is maintained. Recently, Serna-Escolano et al. [22] and Rodríguez-López et al. [23] have proposed the use of essential oils like carvacrol and thymol encapsulated in HP-β-CD to control the in vitro growth of molds and bacteria, respectively. In both papers, we found that the microwave encapsulation method (HP-β-CD-thymol-MW) was the best to encapsulate the essential oils and to control *G. citri-aurantii* growth and its sporulation and bacteria growth. Additionally, Serna-Escolano et al. [22] evaluated the in vitro effect of carvacrol and thymol on *G. citri-aurantii* and 5.06 and 52.6 mM were obtained as minimum inhibitory concentration (MIC) and minimum fungicide concentration (MFC), respectively.

Given the known role of HP-β-CD-thymol-MW in suppressing *G. citri-aurantii* by in vitro experiments, the goal of this research is to assess the in vivo antifungal effect of the HP-β-CD-thymol encapsulated by the microwave method by using lemons inoculated with *G. citri-aurantii*. The curative and preventive effects of the treatment were evaluated as follows. The preventive action is expressed when the antifungal treatment is applied before the pathogen infects the tissues of the plant. The curative action consists in the ability of the fungicide to limit the development of the pathogen inside the tissues, when applied in the latent period, that is, in the interval between the penetration and the appearance of the first symptoms. For this purpose, 50 and 25 mM HP-β-CD-thymol complex concentrations were used, which were close to the minimal fungicidal concentration (MFC) and half of the MFC established in our previous study [22].

## 2. Results and Discussion

**Figure 1:**
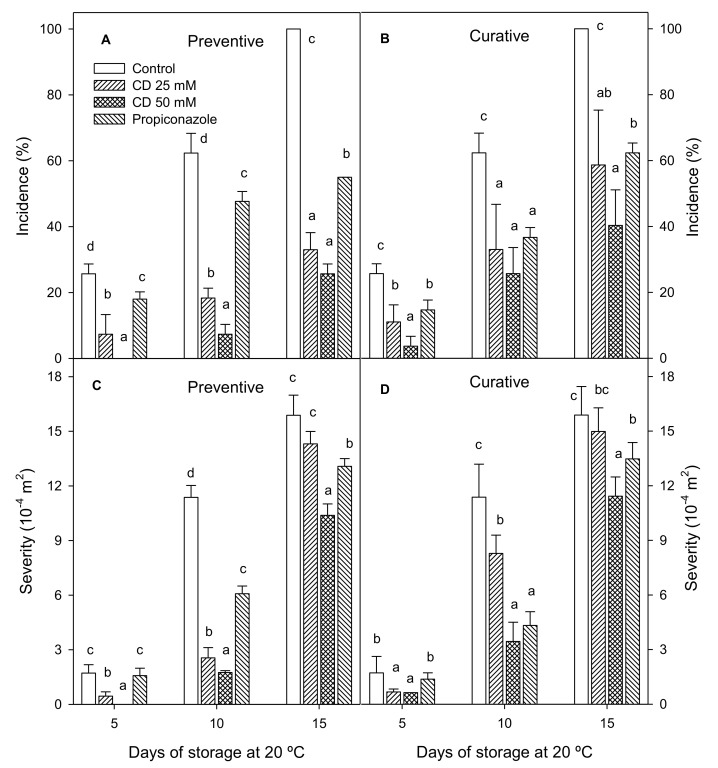
Incidence (%) of fruit affected by *G. citri-aurantii* when 25 or 50 mM 2-hydroxylpropyl-beta-cyclodextrin (HP-β-CD)-thymol or propiconazole were applied as preventive (**A**) or curative (**B**) experiments. Severity (m^2^) of decayed lemons by *G. citri-aurantii* when 25 or 50 mM HP-β-CD-thymol or propiconazole were applied as preventive (**C**) or curative (**D**) experiments. Data are the mean ± SE. Different letters show significant differences (*p* < 0.05) among treatments for each sampling date.

The incidence and severity of sour rot increased significantly throughout storage time in control and treated lemons, although rot incidence in control lemons was always significantly (*p* < 0.05) higher than in treated ones in preventive and curative experiments (Figure 1A,B). Thus, 100% of control fruits were affected after 15 d of storage in the preventive experiment, while fruit treated with 25, 50 mM HP-β-CD-thymol and propiconazole showed a decay incidence of 33.00 ± 5.18; 25.67 ± 2.99 and 55.00 ± 0.01%, respectively. Meanwhile, rot incidence in treated fruit was significantly higher (*p* < 0.05) in the curative than in the preventive experiment with 58.67 ± 16.66, 40.33 ± 10.79 and 62.33 ± 2.99% for 25, 50 mM of HP-β-CD-thymol and propiconazole, respectively. In addition, both concentrations of HP-β-CD-thymol were more effective than propiconazole in decreasing rot incidence in the preventive experiment throughout storage. Moreover, the lowest incidence occurred with the 50-mM treatment. The severity of damage caused by *G. citri-aurantii* during storage was also significantly reduced in fruit treated with HP-β-CD-thymol and propiconazole in both experiments, although the 25 mM HP-β-CD-thymol treatment was similar to control at the last sampling date (Figure 1).

In previous works, the use of thyme oil or thymol, applied directly on the peel of citrus fruit as a coating or in vapor, was able to reduce the growth of different fungi species, such as *Penicillium* spp. [16], *Alternaria* [24] and *G. citri-aurantii* [14]. In these papers, higher essential oil concentrations were used than the thymol encapsulated in HP-β-CD in the present experiment, showing that encapsulation of essential oils increases their effectiveness. Thymol encapsulated in HP-β-CD by MW method is a water-soluble compound and is more stable than that encapsulated in α- or β-CDs [18]. Thymol is slowly released when the HP-β-CD-thymol complex is dissolved in water, and a fungicidal effect on *G. citri-auriantii* without altering the organoleptic properties of lemons is observed. It has been proposed that the mechanism of thymol on inhibiting pathogen growth and spore germination is throughout modifying cell membrane permeability by its direct action on phospholipid and protein degradation as well as on inhibiting ergosterol biosynthesis [25]. Thus, Bagamboula et al. [26] indicated that the presence of a delocalized electron system and hydroxyl groups in the thymol and carvacrol molecules could be responsible for their antifungal activity. Perina et al. [27] demonstrated by image analysis that thymol treatment caused a clear damage on cell wall and plasma membrane disruption, leading to cytoplasm disorganization and organelle death.

On the other hand, Valencia-Chamorro et al. [28] indicated that the antifungal effect of the essential oil depended on whether the applied treatment was curative or preventive. Perina et al. [27] showed that the treatment of thymol prevented fungal penetration and caused a delay in the early infection process of *A. alternate* due to its fungistatic capacity and lack of persistence. This behavior differs from curative commercial fungicides used. Thus, under curative conditions, 10 h of separation between *G. citri-auriantii* inoculation and lemon treatments could be enough for the fungus to invade the fruit cells and HP-β-CD-thymol complex would be less able to control the decay spread. Thus, Sameza et al. [29] reported that infection control becomes difficult once a pathogen penetrates plant tissues.

Montesinos-Herrero and Palou [30] pointed out that the curative or preventive effects of GRAS compounds used in post-harvest disease control depended more on interplay with the host than on chemical fungicide natures used. Moreover, this interaction largely depends on the physical and physiological state of the fruit, as well as on fruit species and cultivars. However, although curative control of the sour rot is not as efficient as preventive control, it is beneficial because it can be applied even if incipient or latent diseases come about.

**Figure 2:**
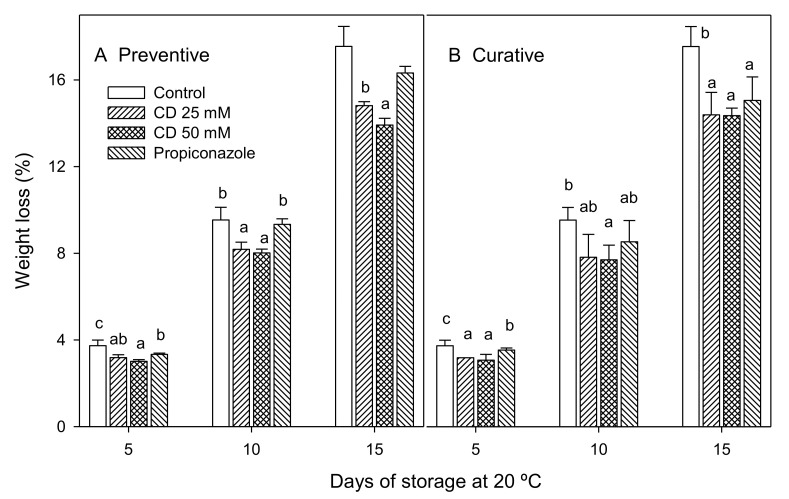
Weight loss (%) of “Fino 49” lemon fruit treated with 25 or 50 mM HP-β-CD-thymol or propiconazole during 15 d of storage at 20 °C in preventive (**A**) and curative (**B**) experiments. Data are the mean ± SE. Different letters show significant differences (*p* < 0.05) among treatments, for each sampling date.

Weight losses increased during the storage of fruit in both assays (Figure 2). However, treated lemons showed significantly (*p* < 0.05) lower weight losses than controls. Treated lemons with 25 and 50 mM of encapsulated thymol in preventive experiment exhibited significantly lower weight loss percentage, with final values of 14.81 ± 0.18% and 13.91 ± 0.31%, respectively, with respect to control and propiconazole-treated fruit (17.55 ± 0.92% and 16.32 ± 0.31%, respectively) (Figure 2A). Weight loss was also reduced in thymol- and propiconazole-treated fruit in curative experiments, although no significant (*p* < 0.05) differences were observed among treatments at the last sampling date (Figure 2B). Lemon weight losses are according to those described in previous papers. It has been observed that the treatments with eugenol, menthol and thymol of cherries [31] and table grapes [32] or the treatments with eucalyptol and cinnamon oil of tomato and strawberry [33] reduced postharvest weight loss of fruit. In addition, mandarins inoculated with *P. digitatum* and *P. italicum* [34] and inoculated oranges with *A. citri* [24] and treated with essential oils from *Zataria multiflora* and *Thymus vulgaris*, showed lower weight losses than control fruit. These effects were related with the reduction of both the incidence and severity of decay, as well as with the reduced metabolism (respiration rate and ethylene production) of the fruit inoculated and treated with essential oils with respect to controls.

**Figure 3:**
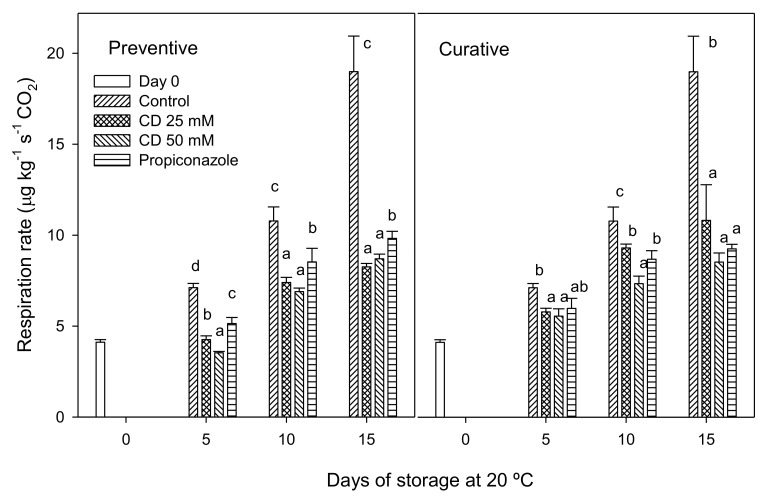
Weight loss (%) of “Fino 49” lemon fruit treated with 25 or 50 mM HP-β-CD-thymol or propiconazole during 15 d of storage at 20 °C in preventive (**A**) and curative (**B**) experiments. Data are the mean ± SE. Different letters show significant differences (*p* < 0.05) among treatments for each sampling date.

The respiration rate of all fruit in both experiments increased throughout storage (Figure 3), although control fruit exhibited the highest respiration rate throughout the experiment, reaching values of 19.00 ± 1.95 µg kg^−1^ s^−1^ after 15 d of storage at 20 °C. However, fruit treated with HP-β-CD-thymol or propiconazole showed significantly (*p* < 0.05) lower respiration rates than control ones, with no significant differences between treated fruit at the end of storage in the curative experiment (~9.2 µg kg^−1^ s^−1^). However, 25 and 50 mM HP-β-CD-thymol-treated fruit showed a lower respiration rate than propiconazole-treated ones in the preventive experiment, 8.25 ± 0.19; 8.70 ± 0.26 and 9.82 ± 0.39 µg kg^−1^ s^−1^, respectively. In addition, treated lemons with 25 and 50 mM of HP-β-CD-thymol in the preventive assay showed a lower respiration rate than in the curative assay. Navarro et al. [17] found in two varieties of nectarine inoculated with three filamentosus fungus species (*B. cinerea*, *P. digitatum* and *R. stolonifer*) that the respiration rate increased exponentially to the volume of damaged flesh tissue in each fruit. In addition, they observed that there was a decrease in rotten fruit percentage and respiration rate after treatments with *Aloe vera* and/or thymol. Similarly, Martínez-Romero et al. [35] found a positive correlation between the increased rotten area caused by *B. cinerea* and ethylene and respiration rate in tables grapes.

**Figure 4:**
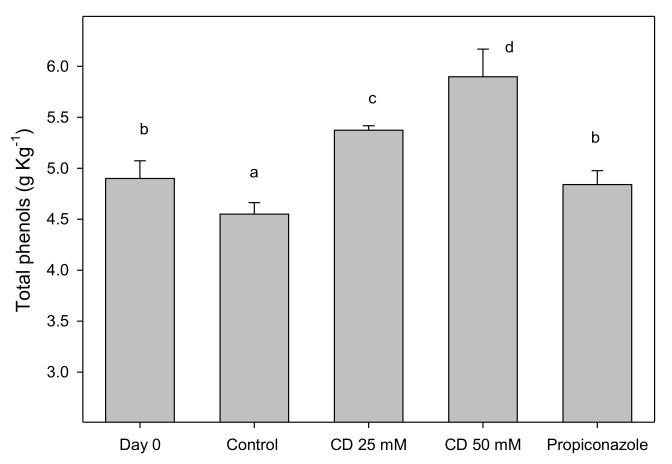
Total phenolics concentration (g kg^−1^ gallic acid equivalent), in peel of control, 25 or 50 mM of HP-β-CD-thymol or propiconazole treated “Fino 49” lemon fruits at harvest (day 0) and after 10 d of storage at 20 °C, in preventive experiment. Data are the mean ± SE. Different letters show significant differences (*p* < 0.05) among treatments after 10 days of storage.

The highest differences in decay incidence and severity, weight losses and fruit respiration rate between lemon fruit treated with 25 or 50 mM of HP-β-CD-thymol and controls were found in the preventive experiment after 10 d of storage. Then, the determinations of total phenolic content and antioxidant enzyme activities (catalase (CAT), peroxidase (POD) and ascorbate peroxidase (APX)) were performed in lemon fruits of the preventive experiment after 10 d of storage at 20 °C. Results showed that total phenolic content in the peel (flavedo + albedo) of control lemons decreased (Figure 4) with respect to values at 0 d by approximately 10% after 10 d of storage at 20 °C. However, in fruit treated with propiconazole no change (*p* < 0.05) in phenolic content occurred, while in lemons treated with 25 and 50 mM of encapsulated thymol the levels of polyphenols increased, reaching values of 5.37 ± 0.04 and 5.89 ± 0.27 g kg^−1^ gallic acid equivalent, respectively. Moreover, the severity of the skin damage of infected fruit was negatively correlated with the polyphenol content, following the semilogarithmic equation log y = −6.03x + log 3.74 (r^2^ = 0.96).

This negative correlation between the severity of the lemon decay and the polyphenol contents could be due to the antifungal capacity of the polyphenolic compounds and the important role that their accumulation in vegetable tissues plays as a protection mechanism against the attack of microorganisms [36]. The initial enzyme implicated in the phenylpropanoid pathway is phenylalanine ammonia-lyase leading to the synthesis of phenolic compounds, which have a pivotal role in defense mechanisms [37]. Bill et al. [38] observed that there was a decrease in the anthracnose severity in avocados treated with chitosan and/or thyme oil coatings, which was attributed to increases in PAL activity, total polyphenol content and antioxidant activity. Thus, the role of encapsulated thymol on decreasing incidence and severity of sour rot could be due, at least partly, to the increased phenolic biosynthesis.

**Figure 5:**
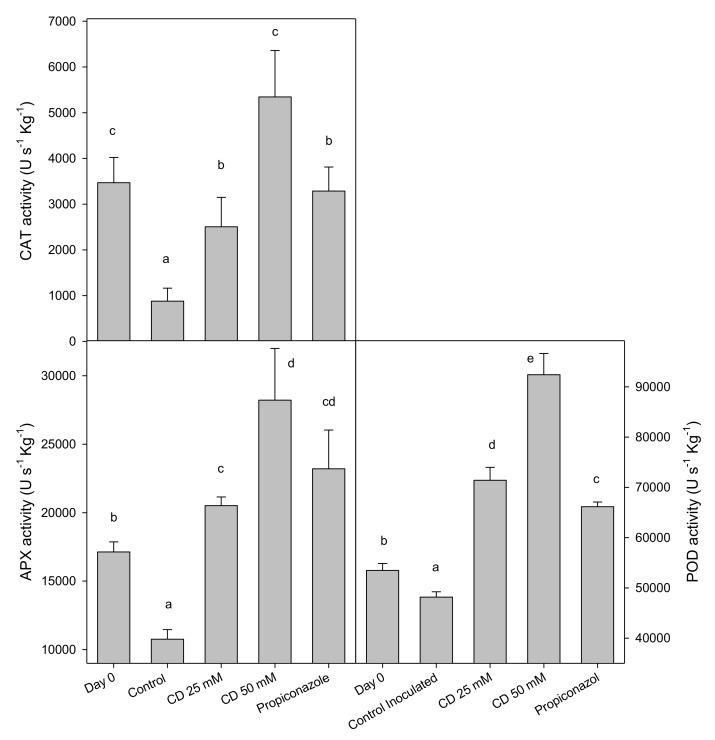
Activity of the antioxidant enzymes catalase (CAT), ascorbate peroxidase (APX) and peroxidase (POD) and, in peel of control and 25 or 50 mM HP-β-CD-thymol and propiconazole treated “Fino 49” lemon fruit at harvest and after 10 d of storage at 20 °C in preventive experiment. Data are the mean ± SE. Different letters show significant differences (*p* < 0.05) among treatments after 10 days of storage.

The activity of antioxidant enzymes (APX, POD and CAT) of control lemons decreased after 10 d of storage at 20 °C compared to activities measured in freshly harvested fruit (0 d) (Figure 5). However, in fruit treated with 25 and 50 mM of HP-β-CD-thymol or propiconazole, these antioxidant enzyme activities increased during storage; the highest values were observed with 50 mM HP-β-CD-thymol treatment for the three enzymes (Figure 5). Decreases in the activity of antioxidant enzymes (CAT, POD and APX) during on-tree lemon fruit development were reported by Serna-Escolano et al. [39]. However, the increased antioxidant enzyme activities found in treated lemons agrees with previous reports, in which treatments of mango [40] and avocados [38] with thymol or thyme essential oils improved antioxidant enzyme activities and free radical scavenging activity and enhanced resistance of fruit tissues against fungal decay. The role of antioxidant enzymes in improving disease resistance in fruit and vegetables has been reported to occur throughout regulation of reactive oxygen species metabolism [36]. Thus, it has been described that SOD activity catalyzes the O^2−^ dismutation reaction into H_2_O_2_ and O_2_ and the enzymes POD and CAT convert H_2_O_2_ into O_2_ and H_2_O [41]. POD activity participates in processes associated with cell wall reinforcement since it is involved in lignin and suberin accumulation which act as defensive physical barrier against pathogenic microorganisms [42]. Therefore, the increase in antioxidant enzymes (SOD, POD and CAT) together with the increased phenolic content induced by HP-β-CD-thymol treatment would cause a delay in the degeneration of cell walls in fruit tissues infected by *G. citri-aurantii* and protect the membrane cellular structure against lipid peroxidation. However, the decrease in antioxidant enzyme activities in control fruit is related with the sour rot severity. All these biochemical modifications would participate in improving the resistance of the peel fruit against the attack of *G. citri-aurantii* and decrease the spread of the sour rot.

## 3. Materials and Methods

### 3.1. Materials

#### 3.1.1. Thymol Encapsulation on HP-β-CD

Thymol (CAS Number 89-83-8) was bought from Sigma (Madrid, Spain). HP-β-CDs (CAS Number 128446-35-5), with an average degree of substitution of 0.5–1.3 units of 2-hydroxypropyl (C_3_H_7_O) for each glucose unit, were provided by AraChem (Tilburg, Holland). The complexation procedure of thymol in HP-β-CDs was fulfilled by using the microwave method (MW) and dehydrated by spray drying [22]. The entrapment efficiency (EE) of thymol complexes in HP-β-CDs was 82.8 ± 0.6. expressed as EE (g 100g^−1^) [22].

#### 3.1.2. Fungus

*G. citri-aurantii* was obtained from decayed lemons as previously reported [22]. Pure cultures of the fungus, in potato dextrose agar (PDA) growth media plates with 6 d of incubation, were suspended with detergent (0.05 Thymol Encapsulated into HP-β-Cyclodextrin as an Alternative to Synthetic Fungicides to Induce Lemon Resistance Against Sour Rot Decay through Increasing Phenolic and Antioxidant Enzymes (Sigma-Aldrich, Madrid, Spain) and sterile distilled water and the arthrospore concentration was tuned to 10^5^ CFU mL^−1^ by employing a hematocytometer according to Serna-Escolano et al. [22] and this inoculum was used in curative and preventive experiments.

#### 3.1.3. Fruit

One thousand mature lemon fruit (*Citrus lemon* cv. Fino 49) were harvested from an organic crop (Mundosol Quality SL Company located in Alicante, Spain) on April, 2019. Then, 720 fruits with homogeneous yellow color and free of visual external damage were selected. Average fruit weight was 0.062 ± 0.005 kg, diameter 0.068 ± 0.001 m, total acidity of 95 ± 4 g citric acid L^−1^ and total soluble solids (TSS) of 103 ± 1 g L^−1^.

### 3.2. Experimental Design

Recently harvested lemons were washed with running water containing 10 µL L^−1^ sodium hypochlorite solution for 3 min. The fruit were dried by forced air at 30 °C for 5 min and housed at random in 24 cardboard boxes with 30 holes. For each treatment, 180 fruit were used, randomly distributed in three replicates. An injury (3 mm width and 3 mm deep) was made on the flavedo and albedo (without reaching the pulp of the fruit) in each fruit using a sterile lancet. Preventive and curative experiments were performed on each half of the damaged fruit. The preventive assay was performed by applying the HP-β-CD-thymol treatment before the inoculation of *G. citri-aurantii*. The curative test was performed by inoculating *G. citri-aurantii* before the treatment with HP-β-CD-thymol. In both assays, between inoculation and treatment application, or vice versa, there were 10 h of separation. Inoculation was performed by adding 40 µL of 10^5^ CFU mL^−1^ conidial suspension to fruit peel wound and HP-β-CD-thymol was applied at two concentrations (50 and 25 mM). In addition, these treatments were compared with control (water), and propiconazole (Bravatia from Tecnidex-Valencia) at a concentration of 1% *v/v*. Two mL of HP-b-CD-thymol or propiconazole treatments were applied by direct spraying on each lemon surface and wounds. After treatment, fruit were stored in darkness at 20 °C and 80% relative humidity throughout the experiment, once they were dried.

After 5, 10 and 15 d of storage, the incidence and severity of fruit damage were evaluated. In addition, each sampling date 5 lemon fruit were taken at random from each replicate, 15 lemon fruit per treatment, to individually analyze weight loss and respiration rate. In lemon from the preventive experiment, a piece of the infected skin surface (flavedo and albedo of 30 × 30 mm) was taken after 10 d of storage in which the infected area was in the middle. The samples were frozen with liquid nitrogen and stored at −20 °C and used to measure total phenolic content and ascorbate peroxidase (APX), catalase (CAT) and peroxidase (POD) antioxidant enzymes activity.

### 3.3. Disease Incidence and Severity

Disease incidence was determined for each sampling day and treatment by enumerating the lemons with sour rot symptoms and using the following formula:

Disease incidence (%) = (decayed fruits/ total evaluated fruits) × 100

Disease severity was determined for each sampling day and treatment by measuring the external diameter of sour rot area of each decayed fruit and the infected area was determined. The data were expressed as m^2^ (mean ± SE).

### 3.4. Fruit Weight Loss and Respiration Rate

The weight losses and respiration rates of lemons were measured individually for each fruit according to Navarro et al. [17]. Weight of lemons was measured at 0 d and after the storage period and weight loss was expressed as percentage with respect to weight at day 0. For respiration rate measures, each lemon fruit was placed in a 0.5-L glass container which was sealed with a rubber stopper for 1800 s. Then, 1 mL of the holder atmosphere was withdrawn and injected into a ShimadzuTM 14A gas chromatograph (Kyoto, Japan), equipped with a molecular sieve 5A column, 80–100 mesh (Carbosieve SII. Supelco Inc., Bellefonte, PA, USA) and a thermal conductivity detector in order to quantify CO_2_ concentration. Respiration rate was expressed as µg CO_2_ kg^−1^ s^−1^ (mean ± SE).

### 3.5. Total Phenolic Quantification

The extraction of total phenolics was performed manually, by homogenizing 2 g of skin tissue samples with 0.01 L of water:methanol (2:8), containing 2 mmol L^–1^ NaF to inactivate poplyphenol oxidase activity, by using a mortar and pestle. The homogenate was centrifuged at 15,000 *g* at 4 °C for 15 min and total phenolics were quantified in the supernatant by using the Folin–Ciocalteu’s reagent as described previously [39]. The results were expressed as g kg^−1^ gallic acid equivalent in a fresh weight basis. A calibration curve was made with gallic acid (Sigma-Aldrich) at concentrations ranging from 5 to 20 10^−9^ kg in the reaction medium, which showed linearity with the absorbance at 760 nm (y = 0.0208x + 0.0045; r^2^ = 0.996).

### 3.6. Activity of the Antioxidant Enzymes

Antioxidant enzymes, such as CAT, APX, and POD were extracted by homogenizing 2 g of flavedo and albedo tissue with 0.01 L of phosphate buffer 50 mmol L^−1^, pH 6.8, containing 1.0 mmol L^−1^ ethylendiamine-tetraacetic acid and 1% (*w/v*) polyvinylpyrrolidone. The extracts were centrifuged at 15,000 *g* for 30 min at 4 °C and the the supernatant was used to measure antioxidant enzyme activities according to our previous report [39]. For APX quantification, the reaction mixture contained 1 × 10^−4^ L of extract in 0.003 L of 50 mmol L^−1^ potassium phosphate (pH 7.0), 1.0 mmol L^−1^ H_2_O_2_ and 0.5 mmol L^−1^ ascorbic acid and the absorbance was measured at 290 nm from time 0 to 60 s expressing APX activity as U s^−1^ kg^−1^, with one enzymatic unit (U) being defined as a 0.01 decrease in absorbance for 60 s^−1^. The reaction mixture to measure POD activity contained 2 × 10^−4^ L of extract in 50 mmol L^−1^ phosphate buffer (pH 7.0), 7 mmol L^−1^ guaiacol and 12 mmol L^−1^ H_2_O_2_ in a final volume of 0.003 L. The absorbance at 470 nm was recorded for 60 s and POD activity was expressed as U s^−1^ kg^−1^, where U was defined as a 0.01 increase in absorbance for 60 s. Finally, to measure CAT activity, 1 × 10^−4^ L of extract was added to 0.003 L of reaction mixture containing 15 mmol L^−1^ H_2_O_2_ in 50 mmol L^−1^ phosphate buffer (pH 7.0). Absorbance at 240 nm was measured at time 0 and after 60 s and CAT activity was expressed as U s^−1^ kg^−1^. U was defined as a decrease of 0.01 in absorbance for 60 s^−1^.

### 3.7. Statistical Analysis

An analysis of variance (ANOVA) was performed by using SPSS 15.0 software version 22.0 (IBM Corp., Armonk, NY, USA). Tukey’s HSD test was used to compare the means considering a statistical significance at *p* < 0.05. The semilogaritmic equations (log y = bx + log bo) were obtained from the severity infection (m^2^) vs. polyphenol contents (g kg^−1^ gallic acid equivalent). The equation fit was quantified by the determination of r^2^ coefficient.

## 4. Conclusions

Thymol encapsulated in HP-β-CD could be an efficient tool as a substitute to synthetic fungicides against *G. citri-auriantii* since its role in reducing the decay incidence and severity in artificially inoculated lemons was even higher than that of the commercial fungicide propiconazole. This effect could be due to its direct antifungal action, its effect of increasing polyphenol content in fruit peel and its action increasing the activity of antioxidant enzymes, leading to increases on cell fruit protection against the attack of this pathogen. In addition, these three effects could be a consequence of the slow liberation of thymol from the HP-β-CD-thymol complex, which in future work could be analyzed.
